# Tracking Openness and Topic Evolution of COVID-19 Publications January 2020-March 2021: Comprehensive Bibliometric and Topic Modeling Analysis

**DOI:** 10.2196/40011

**Published:** 2022-10-03

**Authors:** Maider San Torcuato, Núria Bautista-Puig, Olatz Arrizabalaga, Eva Méndez

**Affiliations:** 1 Innovation Unit Biodonostia Health Research Institute San Sebastián Spain; 2 Library and Information Science Department Universidad Carlos III de Madrid Getafe Spain; 3 Library and Information Science Department Complutense University of Madrid Madrid Spain

**Keywords:** COVID-19, open access, OA, SARS-CoV-2, scholarly communication, topic modeling, research, dissemination, accessibility, scientometry, publications, communication, research topics

## Abstract

**Background:**

The COVID-19 outbreak highlighted the importance of rapid access to research.

**Objective:**

The aim of this study was to investigate research communication related to COVID-19, the level of openness of papers, and the main topics of research into this disease.

**Methods:**

Open access (OA) uptake (typologies, license use) and the topic evolution of publications were analyzed from the start of the pandemic (January 1, 2020) until the end of a year of widespread lockdown (March 1, 2021).

**Results:**

The sample included 95,605 publications; 94.1% were published in an OA form, 44% of which were published as Bronze OA. Among these OA publications, 42% do not have a license, which can limit the number of citations and thus the impact. Using a topic modeling approach, we found that articles in Hybrid and Green OA publications are more focused on patients and their effects, whereas the strategy to combat the pandemic adopted by different countries was the main topic of articles selecting publication via the Gold OA route.

**Conclusions:**

Although OA scientific production has increased, some weaknesses in OA practice, such as lack of licensing or under-researched topics, still hold back its effective use for further research.

## Introduction

### Background

On January 30, 2020, the World Health Organization declared the COVID-19 outbreak a “public health emergency of international concern,” and declared a pandemic on March 11, 2020, at which point the virus had infected more than 150,000 people in 154 countries [1-3]. One year later (March 2021) the number of infected people reached 3.8 million worldwide [4].

The scientific community is facing one of its greatest challenges for research: to quickly develop solutions for the COVID-19 pandemic. This exceptional situation requires a collective scientific effort that has been reflected daily in the publication of hundreds of scientific documents and resources (ranging from articles and reviews to clinical guides or protocols and data). We are likely witnessing the greatest concentration ever of scientific resources specifically directed to the resolution of a common problem [5]. The effectiveness of both the publication system and the different components of traditional scientific communication (journals, databases, and repositories) is crucial to perform medical research as well as other types of research focus (ie, economic, educational, psychological) about this new coronavirus, such as delineating risk factors, clinical features, and treatment strategies, including vaccines [6].

**Figure 1:**
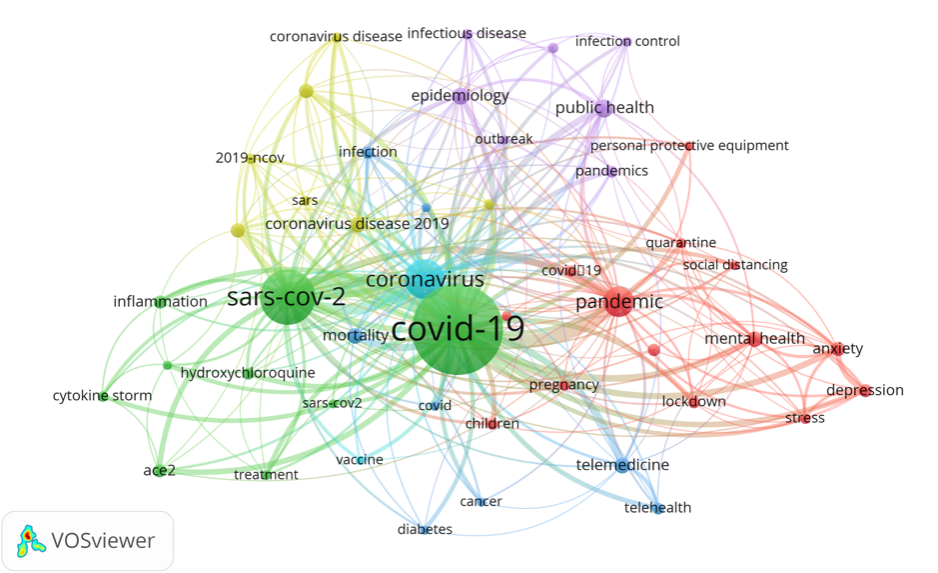
Co-occurrence map within the 50 most frequent keywords among cited SARS CoV-2–related publications with at least 200 publications (data extracted from PubMed: January 1, 2020, to March 1, 2021). Image created using VOSviewer [7].

Research topics have also rapidly changed during the pandemic, focusing on different areas of interest (Figure 1): COVID-19 and treatment (green cluster), populations at risk (light blue cluster), effects of the pandemic on mental health and impacts of social distancing (red cluster), public health (purple cluster), and coronavirus terms or families (yellow cluster).

We adopted a metaresearch approach to investigate the scholarly communication on this disease, particularly focusing on the open access (OA) uptake, along with the evolution of topics about COVID-19 in different OA publication venues.

### Changes in the Scholarly Publication System

COVID-19 has challenged scientists to overcome the “normal” pace of scholarly communication. The main objection that the current system faced from the beginning of the pandemic is two-fold: science that is closed by default and the overload of articles, with 1000 COVID-19–related publications per week estimated at the beginning of the pandemic in PubMed [5]. As a result, a global health crisis has been readily recognized as an information crisis or “infodemic” [8,9].

During the pandemic, numerous efforts were undertaken to make COVID-19 research publicly available as fast as possible. On January 31, 2020, the Wellcome Trust called on researchers, funders, and journals to share data and make findings immediately available to inform the public health response to this outbreak [10]. Signatories to this statement include relevant publishers (Elsevier, Wiley, Springer, Taylor and Francis, among others). This was also followed by large scientific journals, especially biomedical journals (eg*, JAMA, British Medical Journal [BMJ], Science,* Oxford, Cambridge, or *New England Journal of Medicine*) [5], at least temporarily. However, publishers have not always liberated their copyright licenses, and for those who did, it was mainly as an exceptional practice rather than a change of policy.

New pressures and new opportunities were introduced for the scholarly publishing system [11]. Horbach [12] analyzed 669 articles and found that medical journals had accelerated their publication process (eg, the time between submission and publication decreased on average by 49%). However, some studies show evidence of adverse effects, including unethical practices by predatory journals during the pandemic, reduction of journals’ quality standards, or biases (eg, most of the scientific output has been from Western countries or English-only publishing at the expense of local communities that could have relevant insights on the topic) [12-14].

### State-of-the-Art and Previous Bibliometric Studies

Bibliometric techniques have been used to present an overview of COVID-19 research. Efforts have been made to analyze the coverage of different data sources of COVID-19 publications [15-17], using altmetrics (ie, Wikipedia and Mendeley) [18,19], analyzing the effectiveness and impact of collaboration [20,21], gender differences [22], topic evolution [16,23], scholarly communication flow during this pandemic [24,25], as well as OA of these research outputs[5,15].

Although a high volume of scientific publications are being produced (150,000 peer-reviewed COVID-19 outputs were published in the Dimensions database between January 2020 and April 2021, and 40,000 COVID-19 preprints were posted in this period), the percentage of publications on OA differs from that of databases, with 72.81% in Dimensions and 88.8% in PubMed [5,11,15,26]. The majority of OA publications follow the “Bronze” route and are mainly published without a license (representing 76.4% of all OA papers recorded at early stages of the pandemic in PubMed) [15]. However, most bibliometric studies and OA analyses were performed in the early stages of the pandemic.

As pointed out by Colavizza et al [16], the early stage of pandemic research was dominated by the topic of the coronavirus outbreak. However, in analyzing 27,370 publications by topics using Medical Subject Heading (MeSH) terms in PubMed, Wang and Hong [23] found that epidemiology and public health interventions have gathered the highest attention. Within these categories, the most popular topics were prevention and control of COVID-19, whereas other topics have been less popular, such as drug therapy. However, little is known about the differences in OA typologies or licenses, which could help researchers and scientific policymakers understand and guide the status of COVID-19 research.

Accordingly, the aim of this study was to investigate the research communication about this disease, the level of openness of papers, and the main topics of research. We also were guided by the following research questions: What effect has the emergency situation had on scholarly communication? How have OA publishing models affected citation rates? What effect does the presence of a proper license have on the citation of published papers? How have the topics covered in the publications evolved during the pandemic? Does the OA publishing model have an effect on the analyzed topics?

## Methods

### Sources and Search Strategy

**Figure 2:**
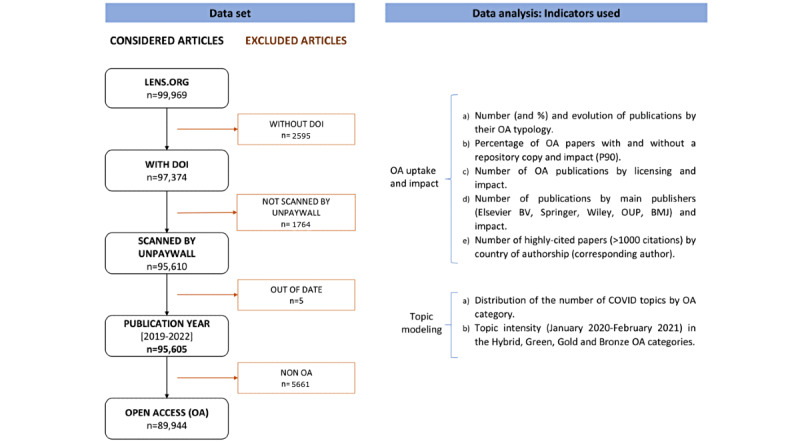
Workflow used to select the sample for the study (sources and indicators).

In this study, different databases and tools were used to collect and analyze COVID-19–related publications, relevant information about OA (typology and licenses), and the main topics covered (Figure 2). The platforms chosen were PubMed, Lens, Microsoft Academics, and Unpaywall that collectively cover a large proportion of free biomedical publications. For this study, we selected PubMed as it is the only database that has been able to record the largest number of publications on this topic since the beginning of the pandemic, including early articles, in an updated manner (daily updating). Other databases such as Web of Science (WoS) or Scopus have a delay of indexing relative to PubMed [15,27]. Furthermore, PubMed is a more well-suited database for biomedical research, whereas Scopus and WoS are more multidisciplinary databases. Moreover, PubMed offers free access to all users, while Scopus and WoS are subscription-based.

The search was performed on March 16, 2021, in the Lens data platform (considering only the PubMed database) by the following query, suggested by the National Library of Medicine and the National Center for Biotechnology Information: 2019-nCoV OR 2019nCoV OR COVID-19 OR SARS-CoV-2 OR (wuhan AND coronavirus)

### Data Selection, Scope of the Study, and Limitations

We focused our analysis on the period from January 1, 2020, to March 1, 2021. This period corresponds with the peak cases in the population and the initial vaccination protocol (immunized) [28]. The query retrieved a total of 99,969 scientific works about COVID-19 in PubMed, 2595 (2.60%) of which did not have a DOI and 1764 (1.76%) of which were not recognized by Unpaywall. Therefore, the study considered a total of 95,605 publications. The Lens database was used to collect 1.6 million citations from the selected publications.

Although this represents a very comprehensive study based on the number of publications analyzed and the different types of analyses performed, some limitations must be pointed out. We only considered one database (PubMed), which is mainly skewed toward medical and biomedical publications and does not cover all academic fields nor all publication languages. Non-English publications and nonbiomedical fields are not covered or are under-represented. Another limitation is due to the use of Unpaywall; although this source provides relevant information on OA, it does not have full coverage and sometimes contradicts information in Crossref. Limitations of the search strategy adopted include the use of the keywords for selecting each COVID-19–related article, which conflicts with the contribution of research toward the pandemic and other studies that might presumably include buzzwords.

### Data Analysis and Research Steps

We first analyzed the uptake of OA and its impact on scientific publications about COVID-19 during the study period (January 2020-March 2021). Figure 2 summarizes the main indicators analyzed. OA status information was considered because OA aims to maximize access to research by promoting visibility and diffusion of scientific outputs and removing technical or financial barriers [29]. Different OA categories defined by Unpaywall were considered in our analysis: Bronze (articles freely available on websites hosted by their publisher, either immediately or following an embargo, but are not formally licensed for reuse), Gold (articles in fully accessible OA journals by paying a fee, known as an article processing charge [APC]), Green (a copy archived in an online open repository with access to final versions after an embargo period), and Hybrid (articles in a subscription journal made OA by paying the APC). In addition, the total number of citations per article, according to Lens, was considered and analyzed by OA typology. However, considering that a skewed distribution is associated with a risk that the citation statistics are dominated by a few highly cited or uncited papers (eg, published in a short time window), a percentile-based bibliometric indicator is needed. Therefore, in this study, we used the 90th percentile (P90) based on total citations received by each paper, which enabled better cross-OA comparisons of the impact of publications. P90 means that the paper belongs to the top 10% most frequently cited papers, which was calculated using linear interpolation of modes in a spreadsheet.

We also used Unpaywall to collect information about licenses. The main licensing options analyzed were Creative Commons (CC) or publisher-specific licenses. Classified according to their level of reuse, from the most open to the most restrictive, the license types include: American Chemical Society (ACS)-Specific, CC, CC-BY, CC-BY-NC, CC-BY-NC-ND, CC-BY-NC-SA, CC-BY-ND, CC-BY-SA, Elsevier-Specific, Implied-OA, PD, publisher-specific license, and no license. In addition, the publisher information was retrieved by analyzing the five most frequent publishers (Elsevier BV, Wiley, Oxford University Press [OUP], and BMJ). Openrefine was chosen to organize, clean up, and analyze the data. This tool allowed us to filter the data extracted from Lens, connect the data with the Unpaywall application programming interface, and to gather more information about OA and the repositories (PMC or institutional repositories found in Open Archives Initiative-Protocol for Metadata Harvesting [OAI-PMH]). For data analysis, interpretation and visualization of a spreadsheet were also used. We further mapped the country distribution of the corresponding author from 105 highly cited papers (with more than 1000 citations, representing 0.11% of the total) using ArcGIS software.

Next, we applied a topic modeling technique to the titles and abstracts of COVID-19 publications by OA types (Bronze, Gold, Green, and Hybrid) to identify prominent topics during the pandemic and their evolution. This probabilistic technique takes a collection of texts as input and makes it possible to identify and learn “topics” from a corpus of documents [30,31]. The keywords from all documents were then grouped by those that appear closer together (by frequency); thus, it can be argued that they are thematically connected, forming clusters (or topics). As a result of this technique, the biggest cluster in Bronze was composed of keywords such as student, medical, or survey, among others, which constituted cluster 0 (see the full list of clusters in Multimedia Appendix 1).

Unlike clustering, topic modeling assumes that each document will fit into one or more topics. Elimination of stop words, spaces, and other irrelevant characters was performed in R software using the tm package [32,33]. A total of 87,744 papers (87.8%) of the data set were used in this analysis. For topic modeling, we adapted Colavizza et al’s [16] code in Open Jupyter Notebook by training the data set with the latent Dirichlet allocation model using the gensim implementation [16,31,34]. In this case, 15 clusters were defined for the identification of keywords divided by OA type, each composed of a group of keywords (see the full list in Multimedia Appendix 1). To more deeply analyze the content, each cluster was categorized into the main topics defined by Colavizza et al [16] and Wang and Hong [23], as described below. “Coronavirus Outbreaks” and “Epidemics” were merged into a single topic (labeled “Epidemics”) as they included similar clusters. The 5 topics and their scope are defined in Table 1. A comprehensive list of topics and clusters is provided in Multimedia Appendix 1.

In addition to this classification, the monthly topic intensity of the clusters (based on the number of publications) by OA type was analyzed to observe the changes over time. As the period of study covered up to March 1, 2021, March was not included in this analysis.

The data set used in this study has been made available in Zenodo [35].

**Table 1:** Topic description and examples of identified keywords.

Topic	Definition and scope	Examples of keywords
Clinical Medicine	Study and practice of medicine that is founded on the direct observation of patients	treatment, chest, therapy, symptom, clinical trial
Immunology	Covers the study of immune systems in all organisms	immune, antibody, drug, vaccine, spike
Molecular Biology	Branch of biology dealing with the structure and function of the macromolecules essential to life	proteins, nucleic acids, virus cell, antibodies, cytokine
Public Health	Branch of medicine dealing with public health, including hygiene, epidemiology, and disease prevention	public health system, patient, mental health, community, nursing
Epidemiology	Studies the rapid spread of disease to a large number of people in a given population within a short period of time	disease, outbreak, countries, masks, tests

## Results

### OA Uptake

#### Overview

**Figure 3:**
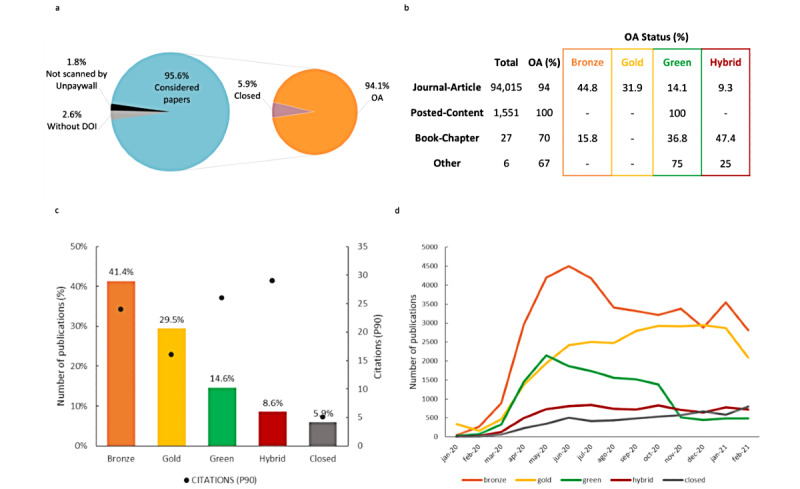
PubMed-hosted SARS CoV-2–related papers published from January 1, 2020, to March 1, 2021 and their open access (OA) status based on Unpaywall. (a) Percentage of considered and excluded papers (without DOI and not scanned by Unpaywall) and their OA ratios. (b) PubMed established publication type and their OA type. (c) Percentage of publications and citations divided by their OA publishing model. (d) Evolution of publications according to their OA publishing model. P90: 90th percentile.

From the 95,605 PubMed articles considered (Figure 2), 98.34% (n=94,015) were journal articles and 94.08% (n=89,944) were published in OA format, with the majority in Bronze OA (44.8%), followed by Gold (31.9%), Green (14.1%), and Hybrid (9.3%) (Figure 3a).

The remaining publications represent posted content (n=1551), book chapters (n=27), and “others” (n=6, including 1 report, 1 peer review, 2 proceeding articles, and 1 uncategorized type) (Figure 3b).

Overall, 41.39% (39,573/95,605) of all publications were published under the Bronze OA model, 29.49% (28,192/95,602) as Gold, 14.64% (13,993/95,605) as Green, and 8.56% (8186/95,605) as Hybrid OA (Figure 3c).

Measuring the P90 of the citation distribution of the field showed that Hybrid, Green, and Bronze OA articles have higher citation values of 29, 26, and 24, respectively, compared to Gold OA articles (16) and articles published in closed journals (5).

Analysis of the evolution of publishing models (Figure 3d) showed that use of the Green model exhibited a decreasing trend during the pandemic, eventually becoming the least-used model. As the pandemic progressed, Bronze and Gold publishing models became more prominent, with a significant increase of the Bronze model from the second quarter of 2020 onward.

**Figure 4:**
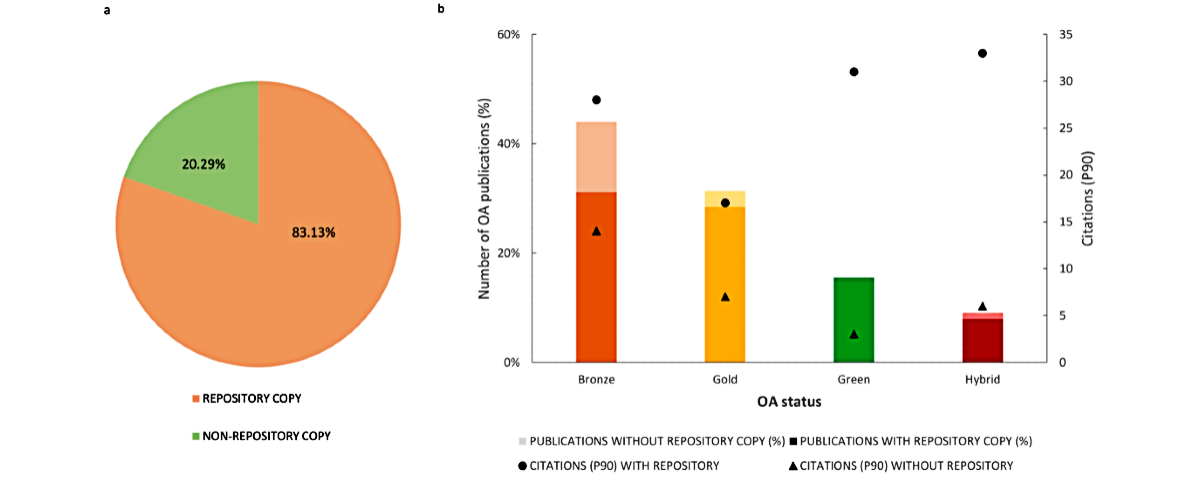
Effect of having a repository copy of open access (OA) SARS-CoV-2–related papers hosted in PubMed (January 1, 2020, to March 1, 2021) on the citations (based on the 90th percentile [P90]). (a) Percentage of OA papers with and without a repository copy. (b) Top 10% of papers with and without a repository copy by OA type.

Figure 4 shows the effect of having a repository copy of OA SARS-CoV-2–related papers on citations. As shown in Figure 4a, 83.1% of the OA papers had at least one copy in a repository (70.7% of Bronze OA; 90.7% of Gold OA; 99.9% of Green OA, although one paper was categorized as Green without a repository copy; and 88% of Hybrid OA publications). Among these papers, 37.4% (n=27,990) were categorized as Bronze OA, 34.2% (n=25,583) as Gold OA, 18.7% (n=13,992) as Green OA, and 9.6% (n=7207) as Hybrid OA. More concretely, in every OA typology, the P90 was higher in the group of publications with a repository copy than in the group of those without such a copy: 28 versus 14 for Bronze papers, 17 versus 7 for Gold papers, 31 versus 3 for Green papers, and 33 versus 6 for papers published in Hybrid journals (Figure 4b).

#### Licenses

**Figure 5:**
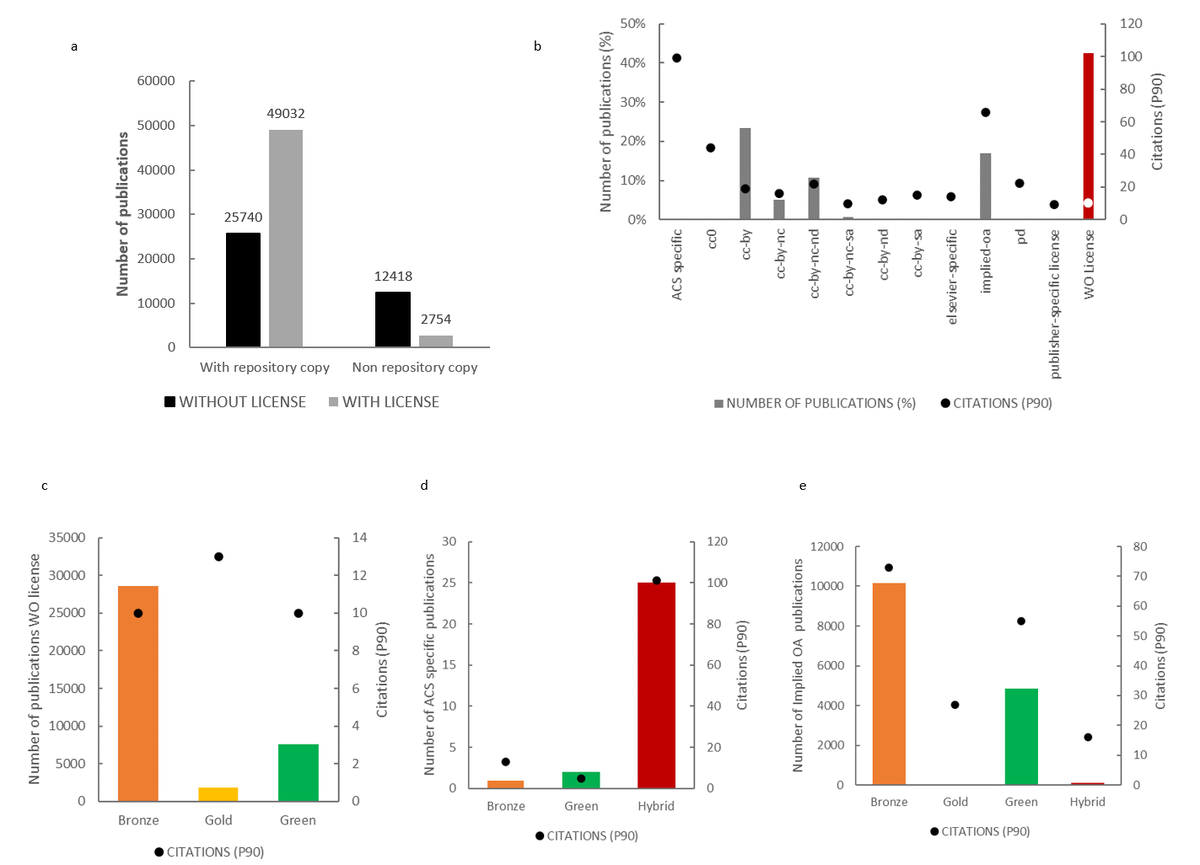
Licensing of open access (OA) SARS-CoV-2–related papers hosted in PubMed (January 1, 2020, to March 1, 2021). (a) Number of papers with and without (WO) a specific licence distributed by OA/non-OA and with/without a repository copy. (b) Distribution of papers based on the licence category. (c-e) P90 and OA status of nonlicensed papers (c), ACS-specific licensed papers (d), and implied OA licensed papers (e). P90: 90th percentile; ACS: American Chemical Society.

We also reviewed the reuse permissions by licenses held by the OA papers: 34.4% (n=25,740) of the papers with a repository copy did not have an explicit license, compared to 81.8% (n=12,418) of those without a repository copy (Figure 5a).

Figure 5b shows that a very relevant number of all OA articles lack a proper license (42.4%), which means licenses allowing free reusability of the paper. The most used licenses are CC-BY (23.3%), followed by Implied-OA (16.9%), CC-BY-NC-ND (10.8%), and CC-BY-NC (5.1%). When the citations of these groups were analyzed, we observed that the highest citation indicator was for papers under ACS-Specific licenses (with 99.1 citations) and Implied-OA licenses (66 citations). Articles without an explicit license showed a poor number of citations (10). Based on these results, these three groups (nonlicensed, ACS-Specific licensed, and Implied-OA licensed) were further studied. For the nonlicensed OA papers, the predominant OA status was Bronze, accounting for 75.1% (n=28,584) of papers with a P90 of 10, followed by Green (20%, P90=10) and Gold (4.9%, P90=13) (Figure 5c). The most cited papers by license type, ACS-Specific licensed papers, were further analyzed. In this case, almost 90% of the papers belonged to the Hybrid OA category with a remarkable P90 value of 101.2 (Figure 5d). Finally, 67.2% of the Implied-OA licensed papers had a Bronze OA status with a P90 value of 73 (Figure 5e).

#### Publishers

**Figure 6:**
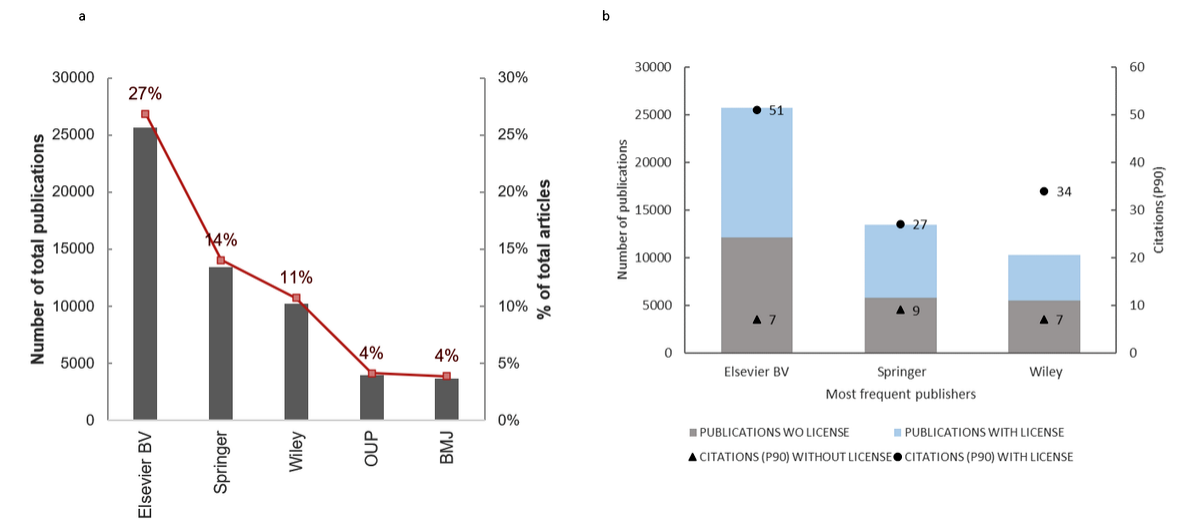
Publishers and journals that published the highest number of COVID-19–related papers hosted by PubMed from January 1, 2020, to March 1, 2021. (a) Number and percentage of total publications distributed by the most frequent publishers. (b) Citation (P90) and presence/absence of a proper licence of all the papers published in the three main publishers. BMJ: British Medical Journal; OUP: Oxford University Press; P90: 90th percentile; WO: without.

The most frequent publisher was Elsevier, publishing 26.88% (25,694/95,605) of the included papers, followed by Wiley (13,461/95,605, 14.08%), Springer (10,266/95,605, 10.74%), OUP (3940/95,605, 4.12%), and BMJ (3701/95,605, 3.87%) (Figure 6a). The presence or absence of a certain license for these publishers was studied in greater depth, as well as the citations (P90) of all the publications published by the three top publishers (Figure 6b). The results showed that 47% (n=12,090) of the Elsevier-published papers do not have a license, and the associated number of citations is low (n=7). However, articles from this publisher with a license had a much higher citation P90 of 51. The same pattern was observed for the next two most frequent publishers: 43% of Springer’s articles do not have any license and their citation level is low compared to the licensed papers (9 vs 27); 53% of Wiley’s papers lack a license and with only 7 citations compared to the 34 citations of the licensed papers.

#### Highly Cited Papers by Country

**Figure 7:**
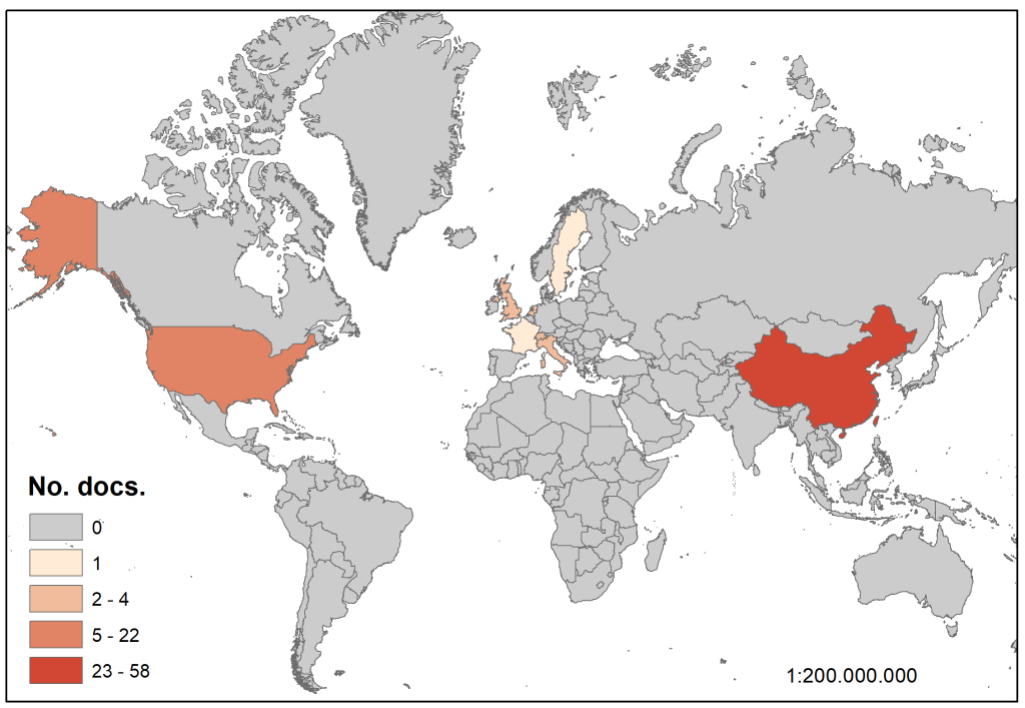
Map of highly cited papers by country of authorship (corresponding author). Image created using ArcGIS [36].

For papers with more than 1000 citations (105 highly cited papers), we determined the country of the corresponding author. China was the country with the most cited papers, including 58 articles with more than 1000 citations (Figure 7). The mean citation value of these 58 papers was 3932, with the highest being 16,164 citations. The two countries with the most highly cited papers were the United States and the United Kingdom, having 22 and 11 papers with more than 1000 citations, respectively. After these three, other countries presented a significantly lower (less than 5) number of highly cited papers (eg, Germany, 4; Italy, the Netherlands, and Switzerland, 2; and France, Singapore, Sweden, and Taiwan, 1).

### Identifying and Monitoring Topic Evolution

**Figure 8:**
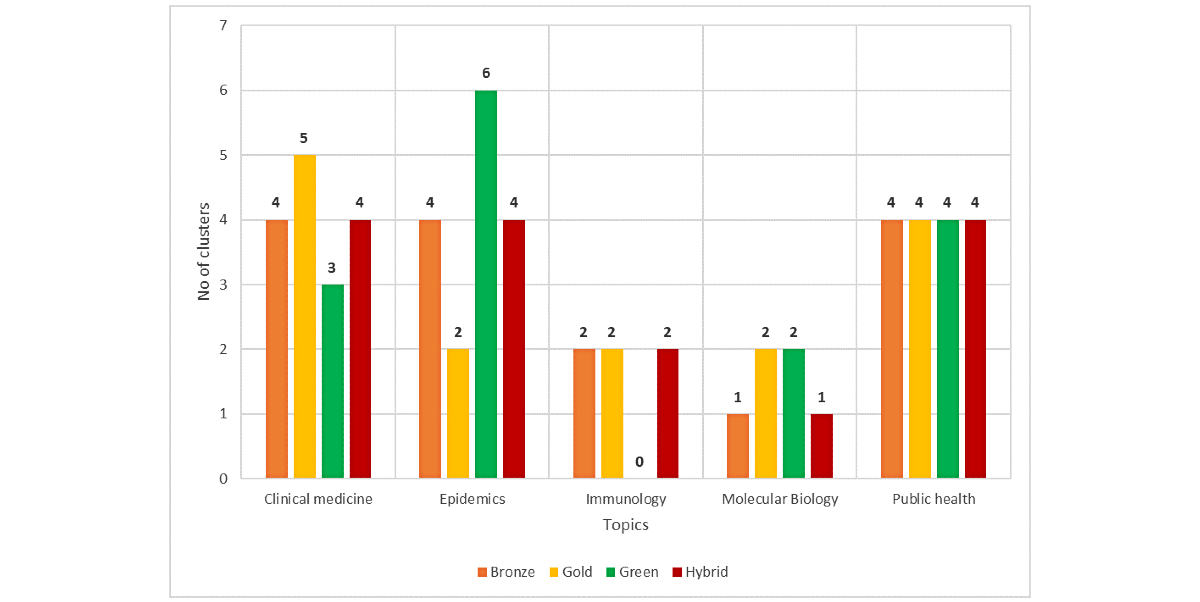
Distribution of the number of COVID-19–related topics by open access type.

A topic modeling technique based on title and abstracts was used to analyze the biomedical content of each publication together with their distribution during the period studied. Figure 8 shows the number of times that each topic was mentioned by thematic cluster and OA category. Topics such as Public Health, Epidemics (ie, monitoring of COVID-19 within countries), and Clinical Medicine (ie, patients, analysis, therapy) were the most frequently addressed, suggesting that the prevention and control of COVID-19 are the most concerning issues at all stages (see Multimedia Appendix 1). By contrast, Immunology (ie, trials and vaccinations) and Molecular Biology (ie, proteins, antibodies) for the purpose of detection and prevention do not exhibit as much interest. Moreover, some topics show a marked preference for specific OA categories, such as Clinical Medicine in Gold OA and Epidemics in Green OA.

**Figure 9:**
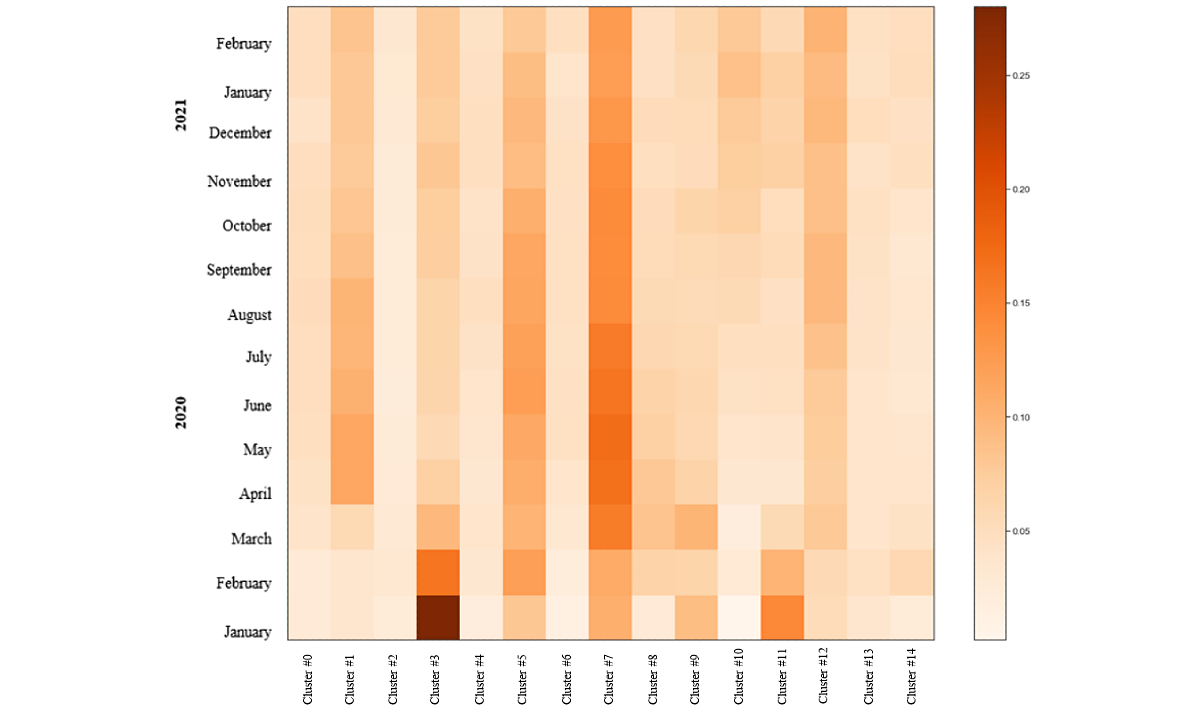
Topic intensity in the Bronze open access journals (January 1, 2020, to March 1, 2021) (n=38,625).

Among the Bronze OA publications, as represented in Figure 9, cluster 7 (health care and services) stood out from March 2020. Cluster 3, terms associated with the lockdown and cases (epidemics), was common in January 2020 but decreased over the course of the pandemic. Another prominent cluster was cluster 5, represented by symptoms (eg, respiratory syndrome), which was more common from February 2020 and this popularity was maintained throughout the study period. Similarly, cluster 1, related to general research on COVID-19 (surveys, interviews, etc), gained popularity from April 2020. With a different pattern, cluster 11 (drugs, protein, virus) was relatively common in January 2020 but decreased over the period of study. By contrast, there were some topics with less presence, including clusters 2 and 6, represented by clinical medicine (eg, pregnant women); cluster 4, represented by immunology; and clusters 13 and 14, represented by epidemics (eg, tests and prediction models).

**Figure 10:**
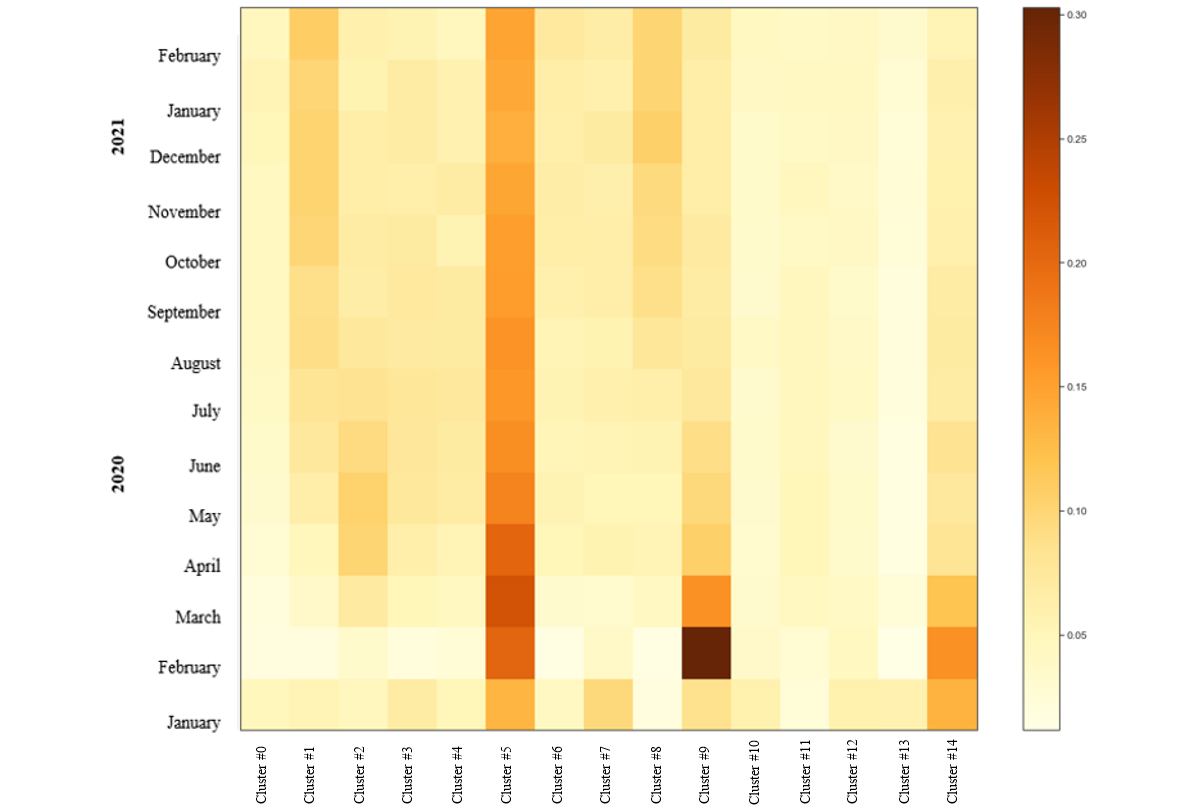
Topic intensity in the Gold open access journals (January 1, 2020, to March 1, 2021) (n=27,786).

Figure 10 shows the evolution of the topics of Gold OA publications. Cluster 5, related to strategies adopted by countries, stood out throughout the period analyzed. Another relevant topic was the number of cases in China (especially during February 2020) (cluster 9) and clinical symptoms (infection, respiratory syndrome) (cluster 14) during the first months of the pandemic (January-March 2020). Cluster 1 and cluster 8, representing clinical medicine (eg, proteins) and public health (eg, mental health effects of the pandemic), respectively, showed a modest increase during the later months of the study.

**Figure 11:**
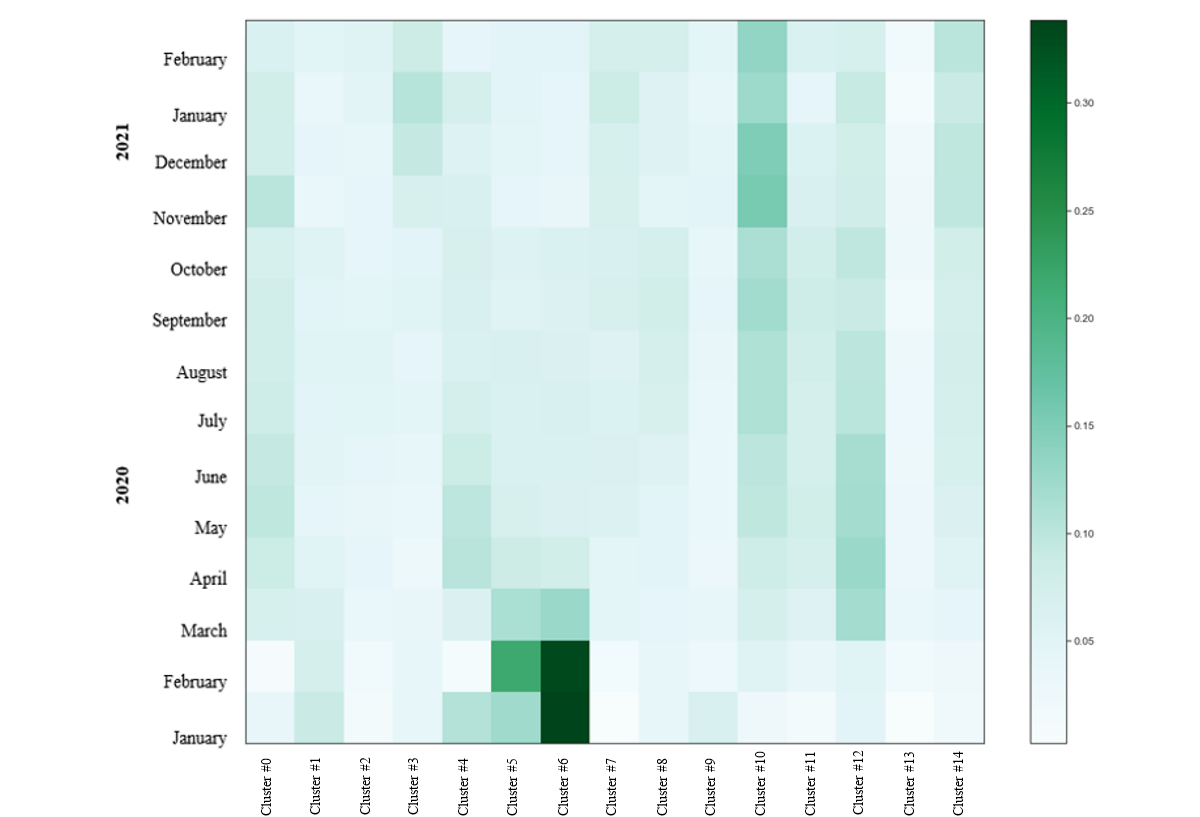
Topic intensity in Green open access journals (January 1, 2020, to March 1, 2021) (n=13,396).

Green OA publications are shown in Figure 11. Topics reflected in cluster 6, associated with respiratory symptoms, were very common in January and February 2020. Cluster 5 (treatments for COVID-19, such as hydroxychloroquine) was strong in February 2020. Other evolutions of interest included patients and hospitalization (cluster 10), which gained relevance over time (notably November-December 2021), whereas treatment (cluster 12; eg, drugs, proteins, and antivirals) started being relevant from March to July 2020 and then interest subsequently decreased. Effects (cluster 2; eg, dental, sleep quality) or symptoms and global measures adopted to prevent the virus (cluster 13; eg, lockdown, social distancing) exhibited relatively less interest.

**Figure 12:**
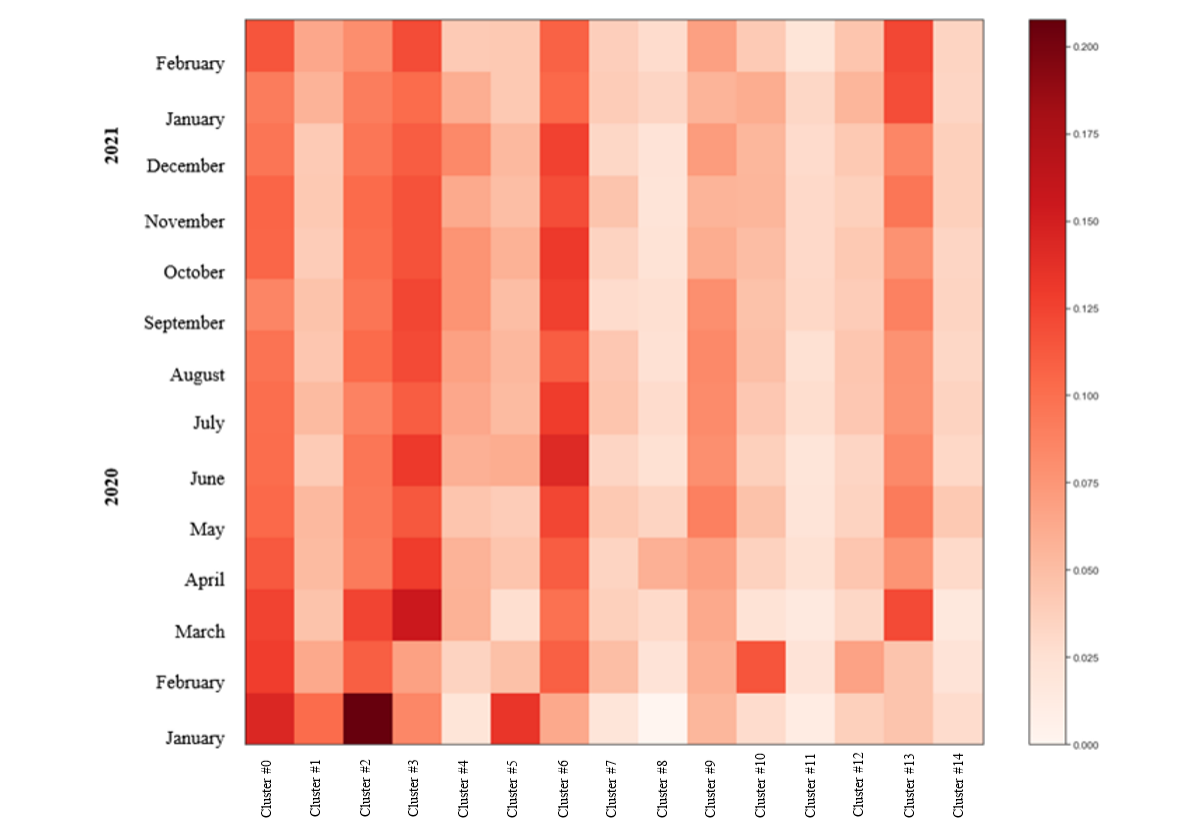
Topic intensity in the Hybrid journals (January 1, 2020, to March 1, 2021) (n=7937).

Figure 12 shows the cluster intensity based on the number of Hybrid OA publications over the study period. Clusters 0, 2, and 5 were the most highly studied topics at the beginning of the period analyzed, corresponding to Public Health and Epidemics. As an example, cluster 2 starts with a burst in January 2020 due to the effects of COVID-19 on psychological and mental health (eg, depression, anxiety, psychological effect) of the population. Notably, clusters 3, 6, and 13, associated with the topics Public Health, Clinical Medicine, and Epidemics, respectively, gained intensity over time. Other clusters showing almost no interest were those related with nursing and care (cluster 8), mortality (cluster 11), and child response (cluster 14).

## Discussion

Based on the large increase in the number of publications during the pandemic [15], the data analyzed in this study (95,605 publications) show that the majority of papers are openly available (94.1%), which is a significantly higher rate than found in other databases (eg, 68% in Dimensions, as pointed out by Torres-Salinas et al [5]). Bronze OA was the most common category, which means that paid journals are providing free access for these publications. The same pattern is also supported by previous studies in different databases such as WoS, Scopus, and Dimensions [5,15,37,38]. Analysis of the evolution of the publications and OA types over time showed that, although an increasing tendency is observed in all OA types, Green OA articles decreased in favor of Gold OA journals during the pandemic, in line with the findings of Nane et al [11].

These results highlighted that the OA impact (measured by the P90) is higher in papers with a repository copy; however, 42% of those OA papers do not have a license, which might be correlated with less visibility and could affect the reuse of the findings. Although the most used licenses are CC-BY, Implied-OA, and CC-BY-NC-ND, ACS-Specific and Implied-OA licenses are associated with a higher number of citations. In this regard, if the knowledge and discoveries are not properly shared and transmitted, the struggle against disease is slowed, with more pronounced fatal effects.

The topic modeling analysis showed that the majority of publications in PubMed focus on Public Health, Epidemics, and Clinical Medicine, whereas Immunology and Molecular Biology are the least addressed topics (complementing the findings of Colavizza et al [16] and Wang and Hong [23]). However, topics such as Public Health and Clinical Medicine play a pivotal role (supporting Wang and Hong [23]), providing new insights to those offered by Colavizza et al [16] on the variation on topics in this specific database.

COVID-19 research topics are continuously evolving along with evolution of their publication trends. Overall, prevention and control are the most prevalent topics (in line with Wang and Hong [23]), while prediction (eg, models to forecast) or treatment (eg, drug treatment), or the effects on specific populations (eg, child response, pregnant women) are the least researched topics. The topic intensity over the months of this study presented different behaviors by OA category. Hybrid and Green OA publications are more focused on the patients and their effects, whereas the strategy adopted by different countries is more frequently published in Gold OA journals, and Healthcare and Services topics are largely published in Bronze OA journals. Although the research focus at the beginning of the pandemic was largely concentrated on disease symptoms or treatments to control the spread of the virus (published in Green, Hybrid, and Gold journals), tests or samples (Hybrid), or the number of cases (Gold)—and these topics prevail continuously, such as the public health system in Hybrid journals or strategies from countries in Gold journals—more recently, the focus has been on the cases by country (Hybrid), patients and hospitalization (Green), or proteins (Gold), among others.

The main conclusions of this study can be summarized as follows. First, the number of COVID-19–related articles in PubMed 1 year following the first global lockdown is 17-times higher than that at the initial stage of the pandemic. This provides new insights into the study of Torres-Salinas et al [5], which estimated a total of 1000 documents per week in PubMed at the beginning of the pandemic.

Second, to effectively confront the global pandemic, we need to make research, and its outcomes, more open. This is an opportunity to show how the scholarly communication system can benefit the public. Although a high number of publications are freely available, not all of them are open and reusable. As clearly demonstrated in this study, more effort on public licensing is needed; 42% of the OA papers related to COVID-19 do not have a license, and this is associated with less visibility, especially for Bronze OA publications.

Third, articles with a higher number of citations include those published under journal-imposed licenses that specify that access to these papers is temporary, allowing reuse and analysis for a limited time, or even allowing reading access for a limited time only.

Fourth, as measured by the number of citations, OA categories (specially Hybrid and Green) seem to be associated with a higher impact than closed journals. Even greater impacts are observed with repository copies (especially those with ACS-Specific licenses and Implied-OA licenses).

Fifth, only approximately 100 papers received more than 1000 citations. Papers written in English, from corresponding authors located in developed countries (United States, China, and the United Kingdom) dominate the highly cited papers.

Sixth, Hybrid and Green OA publications are more focused on patients and their effects, whereas the strategy adopted by countries is more prevalent in papers that have chosen the Gold OA route. Health care and services are the most common topics in the papers published in Bronze OA journals.

Finally, prevention and control were the most prevalent topics in the publications analyzed (coronavirus outbreaks/epidemiology and public health). However, research in some topics is still insufficient (eg, effects on some populations such as children or pregnant women), requiring more global research collaborations.

Overall, monitoring and measuring OA and topic evolution will help researchers and scientific policymakers understand the status of COVID-19 research. This information may be useful as a reference guide, to stimulate new ideas and directions of research, and to help in the fight against this pandemic.

## Appendix

Multimedia Appendix 1 Clusters of COVID-19–related publications according to open access type based on topic modeling.

## Abbreviations

ACS: American Chemical Society
APC: article processing charge
BMJ: British Medical Journal
CC: Creative Commons
MeSH: Medical Subject Heading
OA: open access
OIA-PMH: Open Archives Initiative-Protocol for Metadata Harvesting
OUP: Oxford University Press
P90: 90th percentile
WoS: Web of Science
