# Supplementary material for: Tracking Openness and Topic Evolution of COVID-19 Publications January 2020-March 2021: Comprehensive Bibliometric and Topic Modeling Analysis
Source: J Med Internet Res. 2022 Oct 3;24(10):e40011. doi: 10.2196/40011 (PMC9531723; doi:10.2196/40011)
Supplement: Multimedia Appendix 1 [file jmir_v24i9e40011_app1.docx]

**Multimedia Appendix 1**

Comprehensive analysis of the 15 topic clusters addressed by Bronze, Gold, Green, and Hybrid Open Access papers about COVID-19 (January 1, 2020, to March 1, 2021)

| **Cluster number** | **Keywords used by OA type of publication** | | | |
| --- | --- | --- | --- | --- |
|  | **Bronze** | **Gold** | **Green** | **Hybrid** |
| **Cluster #0** | **Public health**  Student; medical; survey; online; education; virtual; practice; university; training; impact; interview; program; clinical; result; resident; experience; time; change; school; remote | **Molecular Biology**  Cell; ace; receptor; expression; protein; human; infection; gene; enzyme; host; entry; lung; role; increase; system; viral; pathway; virus; tissue; level; | **Clinical medicine**  Patient; disease; acute; respiratory; infection; syndrome; severe; review; injury; case; treatment; clinical; complication; cardiac; report; therapy; include; risk; management; stroke | **Public health**  Health; public; system; public health; crisis; healthcare; response; challenge; global; risk; care; community; provide; research; disease; food; policy; air; approach; development |
| **Cluster #1** | **Clinical medicine**  Patient; cancer; hospital; care; surgery; period; emergency; treatment; case; disease; surgical; management; result; stroke; perform; increase; procedure; report; visit; acute | **Clinical medicine**  patient; disease; mortality; risk; outcome; clinical; high; age; severe; group; associate; hospital; admission; factor; cohort; year; severity; compare; level; result | **Clinical medicine**  Drug; treatment; trial; antiviral; therapeutic; potential; clinical; inhibitor; effect; target; compound; activity; hydroxychloroquine; therapy; efficacy; clinical trial; effective; protease; study; treat | **Epidemics/Coronavirus outbreaks**  Respiratory; virus; infection; worker; syndrome; severe; acute; respiratory syndrome; severe acute; disease; healthcare; healthcare worker; transmission; viral; hcw; influenza; risk; rna; sar; cause |
| **Cluster #2** | **Immunology**  Vaccine; woman; trial; vaccination; pregnant; pregnant woman; pregnancy; clinical; clinical trial; delivery; africa; maternal; development; African; mother; safety; efficacy; dose; phase; breast | **Clinical medicine**  Patient; care; treatment; trial; hospital; clinical; cancer; protocol; control; healthcare; group; management; safety; surgery; risk; procedure; infection; medical; include; guideline | **Epidemics/Coronavirus outbreaks**  Lockdown; transmission; air; period; social; distancing; social distancing; social distancing; reduction; reduce; increase; contact; effect; result; impact; infection; measure; case; high; level; change | **Public health**  Health; mental; mental health; social; impact; study; people; anxiety; high; result; increase; survey; risk; psychological; effect, level; child; lockdown; population; depression |
| **Cluster #3** | **Epidemics/Coronavirus outbreaks**  Case; country; lockdown; rate; number; datum; study; measure; spread; outbreak; review; result; effect; analysis; impact; period; disease; increase; population; china | **Clinical medicine**  Patient; disease; severe; treatment; infection; therapy; immune; respiratory; response; syndrome; cytokine; acute; clinical; inflammatory; therapeutic; cause; cell; ard; acute respiratory; storm | **Molecular biology**  Protein; antibody; vaccine; spike; bind; igg; response; virus; spike protein; viral; human; domain; cell; antibodies; infection; rbd; plasma; mutation; receptor; structure | **Public health**  Care; patient; health; clinical; practice; medical; service; impact; study; disease; change; research; recommendation; include; online; emergency; provide; result; survey; surgery |
| **Cluster #4** | **Immunology**  Response; cell; infection; immune; influenza; type; antibody; immunity; diabetes; virus; patient; disease; virus; patient; disease; immune response; severe; individual; viral; diabete; sar; severity; increase | **Clinical medicine**  Review; patient; study; case; report; disease; symptom; search; systematic; clinical; present; include; evidence; systematic review; article; metaanalysis; acute; manifestation; infection; database | **Public Health**  Patient; surgery; cancer; care; surgical; procedure; telemedicine; visit; risk; emergency; hospital; perform; result; management; method; conclusion; period; disease; guideline; treatment | **Clinical medicine**  Mortality; study; age; patient; risk; death; year; outcome; associate; compare; disease; rate; high; datum; include; admission; factor; woman; cohort; result |
| **Cluster #5** | **Clinical medicine**  Disease; respiratory; severe; acute; syndrome; infection; patient; treatment; cause; severe acute; respiratory syndrome; review; clinical; report; case; cytokine; therapy; inflammatory; system; drug | **Public health**  Health; model; public; country; disease; measure; datum; system; spread; public health; social; response; strategy; control; response; strategy; control; outbreak; policy; epidemic; global; impact; approach | **Epidemics/Coronavirus outbreak**  Case; number; epidemic; country; measure; spread; outbreak; control; time; infection; china; estimate; disease; datum; model; population; intervention; transmission; report; early | **Epidemics**  Test; sample; testing; assay; positive; detection; result; viral; respiratory; virus; diagnostic; method; rna; swab; detect; sensitivity; patient; rt pcr; clinical; infection |
| **Cluster #6** | **Clinical medicine**  Patient; treatment; therapy; ventilation; group; trial; receive; kidney; die; day; ill; transplant; plasma; clinical; icu; critically; mechanical; critically ill; care; intensive | **Public health**  Care; health; child; service; lockdown; impact; increase; adult; state; visit; year; age; population; home; health care; old; period; telemedicine; people; access | **Epidemics/Coronavirus outbreaks**  Respiratory; virus; disease; syndrome; acute; severe; cacao; respiratory syndrome; infection; severe acute; novel; review; human; transmission; health; spread; world; global; outbreak; viral | **Clinical medicine**  Patient; disease; treatment; severe; clinical; care; cancer; hospital; case; study; risk; infection; respiratory; outcome; acute; include; result; high; day; group |
| **Cluster #7** | **Public health**  Health; care; challenge; public; service; system; provide; research; response; crisis; healthcare; public health; global; health care; practice; support; disease; impact; community; resource | **Public health**  Student; medical; activity; online; research; education; physical; change; training; experience; university; physical activity; time; program; technology; food; challenge; digital; provide; learn | **Molecular biology**  Cell; infection; ace; immune; response; disease; cytokine; lung; viral; expression; severe; human; receptor; host; gene; protein; increase; inflammatory; level; tissue | **Clinical Medicine**  Symptom; image; patient; disease; chest; infection; pneumonia; case; result; model; study; imaging; test; contact; finding; feature; asymptomatic; diagnosis; method; positive |
| **Cluster #8** | **Epidemics/Coronavirus outbreaks**  Risk; transmission; mask; infection; worker; healthcare; protective; personal; equipment; disease; ppe; healthcare worker; staff; face; control; aerosol; personal protective; spread; virus; measure | **Public health**  Health; mental; anxiety; mental health; psychological; survey; social; high; participant; stress; depression; level; symptom; result; factor; associate; impact; behavior; worker; risk | **Epidemics/Coronavirus outbreaks**  Model; datum; propose; analysis; approach; base; research; method; network; result; develop; system; predict; disease; paper; different; image; provide; tool; future | **Public health**  Care; nursing; fear; healthcare; distress; digital; ethical; palliative; extracorporeal; membrane; patient; contact; home; response; nursing home; app; ethic; moral; palliative care; ecmo |
| **Cluster #9** | **Public health**  People; child; health; death; government; school; community; year; contact; population; England; state; national; home; black; month; group; disparity; live; report | **Epidemics/Coronavirus outbreaks**  Case; rate; number; infection; death; china; report; transmission; outbreak; confirm; datum; disease; estimate; period; epidemic; result; march; woman; high; country | **Epidemics/Coronavirus outbreaks**  Test; sample; testing; detection; positive; viral; rna; assay; result; diagnostic; method; swab; sensitivity; rt pcr; detect; pcr; rapid; negative; clinical; diagnosis | **Clinical medicine**  Disease; infection; respiratory; severe; syndrome; immune; acute; cytokine; patient; system; response; lung;; clinical; cause; inflammation; cell; mechanism; review; inflammatory; effect |
| **Cluster #10** | **Public health**  Health; mental; anxiety; mental health; social; psychological; depression; stress; impact; result; survey; participant; adult; increase; symptom; physical; high; factor; level; effect | **Epidemics/Coronavirus outbreaks**  Test; positive; sample; testing; detection; result; diagnostic; method; viral; swab; rt pcr; negative; rna; chain; reaction; sensitivity; diagnosis; pcr; detect; laboratory | **Public Health**  Patient; disease, clinical; severe; symptom; outcome; case; result; hospital; high; group; mortality; conclusion; admission; day; associate; infection; cohort; risk; hospitalize | **Immunology**  Vaccine; protein; spike; human; virus; viral; bind; spike_protein; antibody; development; interaction; domain; infection; animal; analysis; genome; mutation; response; sequence; host |
| **Cluster #11** | **Molecular biology**  Drug; protein; virus; ace; human; viral; target; bind; antiviral; potential; molecular; cell; spike; receptor; inhibitor; host; interaction; identity; analysis; therapeutic | **Clinical medicine**  Patient; pneumonia; chest; lung; case; respiratory; image; ventilation; hospital; finding; emergency; oxygen; care; pulmonary; compute; tomography; mechanical; disease; stroke; scan | **Public health**  Health; child; mental; anxiety; mental health; symptom; impact; psychological; survey; social; stress; participant; online; result; depression; report; lockdown; physical; perceive; level | **Epidemics/Coronavirus outbreaks**  Der; die; und; mask; aerosol; model; von; respiratory; eine; mit; droplet; particle; face; fur; method; dem; material; des; face mask; bei |
| **Cluster #12** | **Clinical medicine**  Patient; disease; outcome; risk; mortality; age; clinical; high; associate; group; include; severe; symptom; level; year; compare; admission; result; factor; cohort | **Immunology**  Drug; vaccine; protein; antiviral; target; potential; compound; treatment; therapeutic; development; bind; molecular; identify; spike; candidate; effective; inhibitor | **Public health**  Care; health; healthcare; worker; service; challenge; practice; medical; mask; health care; provide; face; hospital; system; response; experience; work; crisis; personal; program | **Molecular Biology**  Cell; drug; ace; antiviral; receptor; inhibitor; target; human; viral; protease; enzyme; expression; treatment; entry; activity; infection; study; potential; virus; inhibit |
| **Cluster #13** | **Epidemics/Coronavirus outbreaks**  Test; positive; testing; detection; sample; result; assay; infection; patient; negative; antibody; swab; rt pcr; igg; respiratory; viral; detect; asymptomatic; method; diagnostic | **Immunology**  Interaction; viral; analysis; antibody; patient; igg; asymptomatic; infection; test; response; assay; symptom; antibodies; immunity; result; individual; positive; igm; serological; vaccination; sample; symptomatic; day | **Clinical medicine**  Sleep; treatment; dental; quality; wastewater; vitamin; disorder; outbreak; oral; substance; life; method; sleep quality; current; include; blood; biosensor; review; opioid; college | **Epidemics/Coronavirus outbreaks**  Case; number; country; disease; measure; model; transmission; lockdown; epidem; spread; infection; datum; outbreak; control; rate; estimate; death; china; impact; period |
| **Cluster #14** | **Epidemics/Coronavirus outbreaks**  Model; method; datum; lung; propose; base; image; result; approach; chest; system; disease; analysis; tool; develop; predict; pneumonia; feature; prediction; different | **Molecular Biology**  Respiratory; virus; infection; disease; syndrome; acute; severe; respiratory syndrome; severe acute; cause; viral; human; influenza; review; genome; sequence; novel; coronavirus; spread; sar | **Epidemics/Coronavirus outbreaks**  Risk; health; state; mortality; high; death; datum; population; increase; age; factor; study; rate; result; associate; united; outcome; include; individual; united states | **Immunology**  Antibody; study; case; infection; child; level; response; igg; pediatric; result; symptom; disease; patient; high; convalescent; test; participant; temperature; plasma; resport |

Note: Topics (in bold) defined from Colavizza et al. (2020)
